# The Trend of Dental Therapeusis

**Published:** 1888-08

**Authors:** W. Xavier Sudduth


### THE TREND OF DENTAL THERAPEUSIS.

BY W. XAVIER SUDDUTH, M. D., D. D. S., F. R. M. S.

READ BEFORE THE PENNSYLVANIA STATE SOCIETY AT PHILADELPHIA, JUNE 5, 1888.

The prevention of disease is the goal to which medicine looks forward. We have not, as yet, attained the desired end, nevertheless we should not despise the grand strides made in that direction during the past decade. The ravages of many of the infectious diseases have been abated. For the first time in the history of nations has that dread scourge, cholera, been headed off in its western course from its birthplace, in India, and so held in check by better hygienic conditions as not to be very greatly feared, even in the provinces where it prevails as a mild epidemic. Typhoid fever is another example of how science has been able to hold in check a most dreaded disease that was formerly said to be epidemic but which has been proven to be of local origin. It is now known to be contagious only by actual contact, and not infectious in the sense in which that term is generally used by the profession. These advances in the prevention of general systemic diseases have been more than equalled in the progress made in the line of antiseptic surgery and the prevention of infection through other channels ; more especially in puerperal or child-bed fever. These changes have been brought about in medicine through the researches in mycology, whereby the etiology of the various diseases has been made known. Given the known cause of disease and its prevention, in many instances, becomes a more or less simple matter. But before these stages of progress were attained, medicine passed through the quagmires of empyricism and doubt, and in many diseases even yet all we can do is to treat the symptoms. In fact, a school of medicine exists that depends entirely upon symptomology for its *pathology* and indications for treatment. The history of dentistry is very similar to that of medicine. It is to-day just stepping out of the "slough of despond" into the light of a more exact science. If we go back over dental literature we will find many queer ideas, queer in the light of our present knowledge, which were presented and taught in our dental colleges. A student's note-book of ten years ago would look very antiquated if published at the present time.

Who has not had this or that grandparent held up to him as an example because he or she lived on the crusts instead of the good soft part of the bread? Who does not remember such sayings as the following: "We are a nation that live on spoon vituals, hence our teeth decay," "Nature preserves only those organs that are of daily use," "Bandage your arm to your side and it will wither," and such like comparisons, putting the teeth on the same line of vitality as the other parts of the body. How long is it since the "vital theory" of decay received its death blow? This theory held sway for many years, and sprung into existence as a result of hard study on the etiology of decay, but its promulgators were handicapped because they did not have the additional light of the cognate sciences as revealed at the present day. It has long been known that decay was the result of acids, but when we tried to produce decay out of the mouth we could not get the same results as in the mouth, hence it was said that a tooth out of the mouth was a dead tooth, and consequently the conditions were not the same. The natural inference was that the vitality of the living tooth modified the action of the acids in the production of decay. For many years it was held that putrefaction was the cause of decay, and out of this belief grew several lines of practice, some of which are followed to the present day.

Arthur and his adherents proposed to prevent decay by creating "self-cleansing surfaces," by wide separations. This most objectionable practice is, I am happy to say, fast passing into disuse. It has always been a mystery to me how any man with any knowledge of the histology of tooth structure could, for a moment, think that it was possible to improve on nature as to the form of the teeth. Others proposed wholesale extraction of bicuspids and first molars. The sad plight in which the patient was left by reason of the breaking up of the articulation, however, deterred many from practicing this form of mutilation. Still others proposed contour fillings—restoration as near to nature as possible, and which was much more successful, in that, tooth surface was not allowed to touch opposing tooth surface. These all, however, belong to the class of mechanical therapeutics.

As an adjutant to therapeutics very good but as a dependence, sure failure in the end, because it is trying to remove the tooth from the cause rather than the cause from the tooth. All these theories had some basis in fact. The crust our grandmothers ate required considerable mastication, which stimulated a free flow of saliva and consequent dilution of the acids of the mouth. The same is accomplished by chewing tobacco, or gum, or even a toothpick.

The universal acid theory, more in consonance with the appearances, but no more correct as regards its application, has been largely disproven. The putrefactive theory has been entirely abandoned since it has been found that teeth may be placed in putrefying mixtures, and allowed to remain for years without the least trace of decay being produced so long as fermentation did not occur.

In late years, however, much light has been thrown upon the real nature of decay by Drs. Miller, Black and some others, including myself. There is another factor at work in the destruction of the tooth besides acid decalcification. The acid can only remove the lime salts. The form of the tooth at least, in so far as the dentine and cement are concerned, remains intact, and being largely organic, some other agent is required for its disintegration. Dr. Miller has demonstrated that the carious fungus develops an inverting ferment which has the power, after the tooth has undergone decalcification, of dissolving the basis substance. These researches of Dr. Miller gave the final blow to the mineral acid theory, and settled the question of the etiology of decay.

While it must be generally admitted that an acid condition of the fluids of the mouth may have a tendency to cause erosion at the gingival margin, or that inflammatory tissues which come in contact with the teeth, as I have so many times pointed out in previous papers, and the secretion of buccal or labial glands, as demonstrated by Dr. Kirk, may and do produce decay, yet by far the greater majority of carious conditions, being localized points of decay, are the result of local agents developed by micro-organisms at the point where the tooth substance is lost. To Dr. Miller belongs the credit of isolating several of these acid producing fungi and determining the particular acid, and also the investing agent produced by them. It has been argued by some that Leber and Rottenstein preceded Dr. Miller in this line of work, while it is true that they and

Magitot as well had worked in the same direction, yet it is also true that they failed to positively prove their positions by isolating the special micro-organism that caused decay. Dr. Miller not only isolated the fungus, but determined the acid developed by it, and produced decay out of the mouth by the direct action of these special organisms that cannot be detected from "natural" decay occurring in the mouth.

I would not detract an iota from the credit due for the work they did, nor do I say that their work, which was in the right direction, did not help Dr. Miller in his researches. I was working on this subject at the same time as Dr. Miller, and made my report to the Illinois State Society in May, 1884. I discovered what Dr. Miller subsequently published, that the micro-organisms, while thickly distributed in the semi-decalcified dentine, did not penetrate the sound, healthy dentine. That was work in the right direction also, but I did not determine the character of the fungus as did Dr. Miller, so I say honor to whom honor is due, and I hope, when Dr. Miller shall visit this country this summer, to see the dental profession give him such an ovation as was never given to any other dentist in this country before. The profession owes it to him, for he has solved the problem of dental caries and has made it possible to formulate an exact line of dental therapeutics that will do more in the end to help reach that desired position, where we can anticipate decay, than all the combined efforts of the profession before him. Having the known etiology of a disease we are in better condition to treat it antiphlogistically. The question naturally arises, how shall we best restore the parts to a better hygienic condition, taking for granted that caries is the result of an unhygienic condition? The answer comes, keep the mouth clean. But the word cleanliness has largely increased its meaning, since so much has been done in the direction of bacteriology.

Formerly everything was clean that we did not know was dirty, now everything is dirty that we do not know to be microscopically clean. The microscope and bacteriological apparatus have been placed side by side with chemical reagents, retort and scales. A mouth may look ever so clean and yet be most foul when judged from our present standard of cleanliness. Dirt is only a matter of comparison. The best definition I ever heard for it was "matter in the wrong place." The soil in the field is all right, but on "my lady's fingers" all wrong, and so it is with micro-organisms; they have their place in nature, or they would not be there, but the mouth is not that place, and when so found they put that cavity in a decidedly unhygienic condition, and it is with such a condition that we propose to deal.

We will first consider how micro-organisms operate deleteriously upon the teeth. I remember a paper that was read by a New York dentist, on the etiology of decay, in 1882, in which he took the ground that these fungi lived upon the lime salts of the teeth, and that instances were on record where teeth had been found honeycombed by their action. This theory has been thoroughly disproven, and it is now positively known that several of the fungi that find a suitable culture media in the oral fluids have the power of producing lactic acid. The Bacillus acidi lactici has long been known to mycologists. Its physiology and morphology have been fully studied in its relation to the souring of milk. It grows in rods of varying lengths, generally from 1—2.8 mm. long, although sometimes found in long mycilial threads. It is from .3—.4 mm. thick — the thickness varies, being thinnest when cultivated upon nutrient gelatine. It does not produce cocci, but forms spores, and grows best at a temperature 39c.–42c. At a temperature exceeding 45.5c. they are no longer capable of producing any activity. It is probable that several forms of micro-organisms have the power of souring milk.

Dr. Miller thinks, however, that they all belong to the same genera, and that the variation is due to the change in conditions under which they are developed. It is possible that he is right in the matter. He has determined several forms in the mouth that have the power of producing lactic acid, and considers them variations of the same fungus. My studies have led me to the conclusion that not much dependence can be put in mere form alone ; we must combine our researches to the physiological action of cells, whether they be of a low or high grade in the scale of existence.

Of the particular species of fungus that produces decay, according to Dr. Miller, the fungus presents as cocci, deplococci and as pronounced bacteria. The variations are so great that unless seen in one chain, as he has often done, he could hardly have believed that they belonged to the same genera. The variations in form account for the presence of the different shaped micro-organisms found in the expanded dentinal canals. Dr. Miller thinks, however, that another genera might be differentiated that presents itself as a bacillus in most instances, but which is also found in the form of Leptothrix, bacteria and cocci; either singly or in zigzag threads. Miller has denominated these two forms as $\alpha$ and $\beta$—Alpha fungus, Beta fungus—and has positively shown that they are both connected with the production of lactic acid.

I am inclined to consider these fungi as different forms of the bacterium which is most commonly found in sour milk—although not altogether agreeing with the description presented by Pasteur. The question as to form and genera is one of minor importance, however. They all act in a similar manner by converting sugar into lactic acid. It does not matter whether the sugar be cane or grape.

A new sugar, saccharine, has been lately discovered that is said to be absolutely non-fermentable, which may in time come to supplant glucose that is now so largely used in the adulterations of our confections. Lactic acid is also produced in the mouth by granulation tissue. Acetic acid and formic acid are also produced by the oxidation of alcohol. The latter, however, in very small quantities, and it is a question whether the generally acid condition of the saliva has any very great decalcifying action on the teeth. I am certain that decay is a local disease, and that the agent is produced at the point of decay in the great majority of cases. Erosions are produced in most instances, however, by the generally acid condition of the saliva. Believing then, as I do, that fully nine-tenths of the decay found in the mouth is caused by a fungus that has its habitat in the oral fluids, it is not strange that I should advocate antiseptic rather than neutralizing agents. Alkalies have only an ephemeral action at best, and are known to have an injurious action on temporary fillings. The most commonly applicable agent is one that will strike at the root of the evil, and by destroying the active agent eliminate decay. This is not always found practicable, however, and in many instances we must content ourselves with preventing the development of the micro-organisms that produce decay.

Dr. Miller did considerable work in the direction of determining the antiseptic value of a long list of drugs, Miller's list included: Bichloride of mercury, Nitrate of silver, Iodoform, Naphthaline, Iodine, Oil of mustard, Permanganate of Pot., Eucalyptus oil,

Carbolic acid, Hydrochloric acid, Phenyic acid, Carbonate of sodium, Salicylic acid, Alcohol.

To these I have added the following, and present to you for examination: Hydronaphthol, Beta Naphthol, Naphthalin, Antiseptic pastiles, Sozojodol, Zinc sozojodol, Sodæ sozojodol, Potass. sozojodol, Murcuric sozojodol, Saccharine, Salol, Silico flouride of Soda, Silico flouride of Zinc and Soda, Salicylate of Zinc, Benzoated sol. alluminium, Liquor carbonic detergent, Labarraque's sol. Boroglyceride, Styrene, Trichlorphenol, Resorsin, Thymol, Subiodide of Bismuth, Boric acid, Alluminium Acitotraticum, Iodol, Borate of Zinc, Chloride of Zinc, Hydrochinine, Permanganate of Zinc, Benzoic acid, Creosote, Aseptol, Sulphocorbolate of Zinc, Peroxide of Hydrogen.

It is my intention to test all these agents, first in the laboratory and then in the mouth, and if possible determine their exact antiseptic value. I shall include in my experiments, also, all the different forms of tooth washes that are on the market and report to the profession at some future date.

